# Validating Sam68 expression and protein level in breast cancer

**DOI:** 10.25122/jml-2022-0229

**Published:** 2023-07

**Authors:** Muneer Kadhum Khudhair, Noaman Ibadi Mohammed, Rahman Sahib Zabibah

**Affiliations:** 1 Department of Laboratory Investigation, Faculty of Science, University of Kufa, Kufa, Iraq; 2 Department of Physiology, Faculty of Veterinary Medicine, University of Kufa, Kufa, Iraq; 3 Medical Laboratory Technology Department, College of Medicine, Islamic University, Najaf, Iraq

**Keywords:** breast cancer, Sam68, breast metastasis, BC: Breast Cancer, ELISA: Enzyme-Linked Immunosorbent Assay, CSC: Cancer Stem Cell

## Abstract

Breast carcinoma ranks as the second most common cancer among women worldwide. Despite significant therapeutic advancements, approximately 25% of breast carcinoma cases have resistance to current treatment modalities, posing a significant challenge for patient management. This study aimed to investigate the role of Sam68 mRNA and its protein in promoting oncogenesis and breast cancer progression. Sam68 protein levels were assessed in tissue samples using an Enzyme-Linked Immunosorbent Assay kit from Sun Long Biotech. Whole RNA was isolated from malignant breast tissue samples obtained from patients. The RNA concentration was determined using an Eppendorf photometer, yielding an average concentration of 62.1±10.07 ng/µl. The purity of the isolated RNA was evaluated by measuring the A260/A280 ratio (1.9±0.07) and the A260/A230 ratio (1.7±0.3). The results indicated a significant upregulation of Sam68 mRNA expression in breast cancer tissues, supporting the findings from previous studies and indicating the correlation between altered Sam68 expression and the development of breast carcinoma, highlighting the potential significance of Sam68 in the pathogenesis of the disease. Estimating Sam68 in the blood may serve as a potential biomarker for assessing the malignant grade and metastatic spread of breast carcinoma cells.

## INTRODUCTION

Breast cancer (BC) is one of the most prevalent and aggressive malignancies affecting women, accounting for approximately 25% of all cancer cases and contributing to 15% of cancer-related deaths [1]. Early detection and timely intervention offer promising chances of successful treatment, with curability rates reaching up to 80%. However, advanced breast cancer represents a great challenge, with average overall survival ranging from two to three years [2]. Among the factors leading to poor outcomes, metastasis is the primary cause of mortality in various cancers, including breast cancer. Despite advancements in early detection methods, metastasis remains a significant challenge in breast cancer management [3].

Recent research has highlighted the role of cancer stem cells (CSCs) as key players in metastasis. These cells have the unique ability to give rise to diverse cell types within a particular tumor, fueling tumor heterogeneity and rendering cancers resistant to conventional treatments. Moreover, CSCs exhibit an enhanced capacity for metastasis, promoting tumor recurrence and progression [3, 4].

Sam68, also known as Src-associated in mitosis of 68 KD, was initially identified as a protein physically associated with phosphorylation via c-Src during mitosis [5]. Sam68 is considered an RNA-binding protein belonging to the STAR (signal transduction and activation of RNA) family that associates cellular signaling to ribonucleic acid transformation and is clearly expressed in tissues and cells of breast cancer. Sam68 has been shown to have a dual function as an RNA-binding protein and a docking protein in various cellular proceedings [5]. The up-regulation of Sam68 is registered in prostate carcinoma and many proliferative lesions of women's breast carcinoma [6]. Moreover, it has been involved in the pathogenesis of several human cancers, including ovarian, breast, and prostate cancer, among others [7, 8]. This study aimed to investigate the role of Sam68 mRNA and its protein in promoting oncogenesis and breast cancer progression.

## MATERIAL AND METHODS

### Sample Collection

This case-control study included paired tissues from 60 female patients (aged 45-65 years) diagnosed with breast carcinoma. Tissue samples were collected from breast carcinoma and adjacent normal breast tissues. All patients were diagnosed with breast carcinoma and underwent surgery at the Department of Surgery of Euphrates Center for Cancerous Tumors in Al-Najaf Al-Ashraf Province between September 2021 and March 2022. The study participants were married Iraqi women, predominantly from middle-income backgrounds. Single women were excluded from the study.

### Breast cancer detection and diagnosis

Breast cancer detection procedures for the study participants involved mammography to identify suspicious lesions and biopsy to histologically confirm the tumor type. Before surgery at the Euphrates Center for Cancerous Tumors, a computerized tomography (CT) scan was performed to determine tumor dimensions and plan excision procedures, ensuring the accurate differentiation of cancerous tissues from healthy ones. Patients were diagnosed with stage I-III breast carcinoma, and no other cancer was diagnosed. In addition, patients did not receive any chemotherapy or radiation before the surgery. The tissue specimens collected during surgery were 3–2 mm^3^.

### Extraction of RNA & qRT-PCR analysis

Total RNA was extracted from the collected tissue samples using the GENEzol™ TriRNA Pure Kit from Gene Aid Company, following the manufacturer's instructions. RNA concentration was confirmed using a NanoDrop™ 1000 Spectrophotometer. Reverse transcription reactions were performed using oligo dT Primers from Thermo Fisher. Quantitative Real-time PCR (qRT-PCR) assays were conducted using a SYBR-green master mix. GAPDH was used as the housekeeping gene (Table 1).

**Table 1:** Primers sequences of qPCR

Gene	F- Primer	R- Primer
Sam68	CTCCGCTAGGCCAGTGAA	TTGTGGGTAAAGCAACAGGA
GAPDH	GACTCATGACCACAGTCCATGC	AGAGGCAGGGATGATGTTCTG

### Determination of Sam68 protein

Sam68 protein levels were quantified in tissues using an Enzyme-Linked Immunosorbent Assay (ELISA) kit from Sun Long Biotech.

## RESULTS

The analysis of RNA concentration revealed an average level of 62.1±10.07 ng/µl in the collected breast tissue samples. RNA purity was assessed by calculating the A260/A280 and A260/A230 ratios, resulting in values of 1.9±0.07 and 1.7±0.3, respectively, confirming the quality of the extracted RNA.

**Figure 1:**
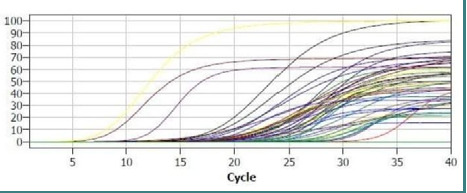
Ct cycle of Sam68 in breast cancer and healthy tissues

The first three curves in Figure 1 represent a positive control related to the DNA of the same gene. Then the six purple curves belong to the endogenous gene, with the subsequent curves representing the studied gene in the study participants' samples. Ct values above 28 were characteristic of healthy tissue samples.

**Figure 2:**
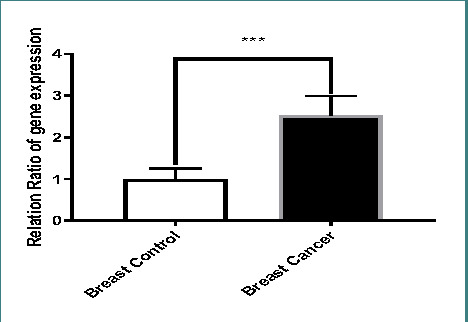
Comparison of Sam68 expression ratio between breast cancer tissues and healthy controls

As shown in Figure 2, the gene expressions of Sam68 were significantly increased (p<0.001) in breast cancer tissues, with an approximately two-fold increase compared to healthy tissues. The t-test results in Table 2 demonstrated a statistically significant difference in mean expression between breast cancer (BCa) tissues and adjacent normal breast tissues.

**Table 2:** Comparison of Sam68 expression levels in breast cancer tissues and healthy controls

Breast Tissue	Control	Carcinoma
Mean	1.000	2.533
Std. Deviation	0.259	0.460
Std. Error of Mean	0.033	0.059
Lower 95% CI	0.9331	2.414
Upper 95% CI	1.067	2.652
p-value	<0.0001	

## DISCUSSION

In this study, we successfully extracted whole RNA from breast carcinoma tissue specimens, allowing us to quantify the gene expression of Sam68 in relation to adjacent normal breast tissue. The use of GAPDH as a housekeeping gene provided a reliable control for relative quantification, as depicted in Figure 1, where Ct values from real-time PCR demonstrated consistency and accuracy.

The study involved paired tissue samples, one obtained from breast carcinoma and the other from adjacent healthy breast tissue, from 60 female patients aged between 45 and 65 years. Approximately 80% of the women in the study belonged to poor to medium living conditions, while the remaining 20% came from affluent backgrounds. Despite the socio-economic disparity, a common factor among all participants was their low level of education and limited knowledge about medical care and health. More than 24% of the patients reported a family history of breast cancer. The racial diversity of the participants was evident as they represented various regions in Iraq. Histopathology diagnosis revealed that ductal carcinoma accounted for 60% of cases, while lobular breast cancer represented 40% of the cases. Triple-negative breast cancer constituted only 5% of the cases, likely due to its higher prevalence in younger women. The majority of cases were categorized as estrogen, progesterone, and Her2-positive.

Our results demonstrated a significant up-regulation of Sam68 gene expression in breast cancer tissues compared to healthy tissues. This observation aligns with previous studies linking Sam68 up-regulation to enhanced proliferation, reproduction, and invasiveness of cancer cells. The results suggest that Sam68 could participate in the spread of breast cancer and have various tumor-promoting roles in several cancers, including breast cancer [9-11]. Studies have also indicated that Sam68 is vital in hormone metabolism, signal transduction pathways, and breast cancer stem cell renewal.

Our findings align with other studies that have reported a highly significant increase in Sam68 expression in breast carcinoma tissues compared to healthy tissues. Elevated Sam68 expression was associated with clinicopathological parameters and prognosis, suggesting its potential role in advancing breast cancer and metastasis [12]. The significant concentration of Sam68 mRNA in breast cells underscores its importance in promoting cellular processes, and its depletion has been linked to decreased breast cancer cell generation and altered neural stem cell recognition [6, 9]. Additionally, a unique splice variant of Sam68 lacking a KH domain (SAM 68-KH) has been associated with cell cycle arrest in fibroblasts [13, 14]. Another study found that Sam68 has an important role in the metabolism of hormones insulin and leptin, as its role appears in the signal transduction pathways of these hormones in three different directions for breast adenocarcinoma cells [16]. Others found that the expression of endogenous Sam68 correlated positively with the regeneration potential of breast cancer cell lines. One study showed that miR-204 regulates the gene and plays a crucial role in the spontaneous renewal of breast cancer stem cells by activating the Wnt/beta-catenin pathway [17].

A recent study showed that NEK2 interacts with riboprotein binding Sam68 in triple-negative breast cancer cells. NEK2 mediates the phosphorylation of SAM68 protein, thus stimulating splicing, indicating that NEK2 and SAM 68 work together in regulating splicing, which sustains the prometastatic features in triple-negative breast cancer cells [18].

**Figure 3:**
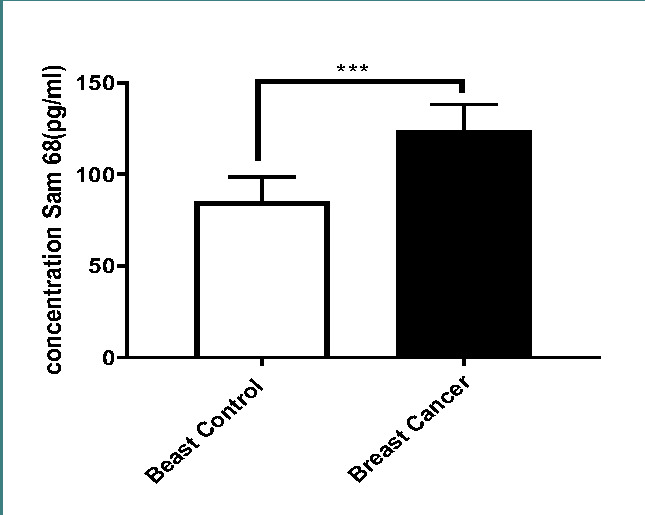
Sam68 protein concentration (pg/ml) in breast cancer tissues

Statistical analysis using the T-Test has confirmed that Sam68 concentration is significantly higher in breast cancer tissues compared to healthy tissues (p<0.001), as depicted in Figure 3 and Table 3. These findings support previous research showing elevated Sam68 expression in breast carcinoma tissues compared to normal tissues [12, 15].

**Table 3:** Comparison of Sam68 protein concentration (pg/ml) between breast cancer tissues and healthy controls

Breast Tissue	Control	Carcinoma
Mean	85.36	124.0
Std. Deviation	13.38	14.14
Std. Error of Mean	1.72	1.82
Lower 95% CI	81.90	120.4
Upper 95% CI	88.82	127.7
p-value	<0.0001	

Based on the results, it was determined that Sam68 up-regulation is a common molecular variant in breast cancer, and there is a close relationship between Sam68 upregulation and tumor growth and progression. The close association between Sam68 up-regulation and tumor growth and progression suggests its potential as a therapeutic target in the management of breast cancer.

## CONCLUSION

Our study revealed a significant increase in Sam68 mRNA expression in breast cancer tissues, consistent with findings from previous research. This observation suggests that altered Sam68 expression is closely associated with the development of breast cancer and may play a crucial role in the pathogenesis of the disease. Although the gene may not be the primary cause of breast cancer initiation and progression, its elevated expression serves as a potential indicator of the presence of a malignant tumor. Sam68 can be detected in the blood, and by assessing its levels in circulating tumor cells, which have migrated into the bloodstream or lymphatics from the tumor, we may indirectly assess the malignant grade and metastatic potential of breast cancer cells. The number of leaked cells in the blood increases with the size of the tumor and its spread.

## Personal thanks

The authors would like to acknowledge the staff at the Faculty of Science, Faculty of Veterinary Medicine, and the College of Medicine, Islamic University, for their invaluable assistance, advice, and technical support throughout this research.
